# Digital breast tomosynthesis for breast cancer screening and diagnosis in women with dense breasts – a systematic review and meta-analysis

**DOI:** 10.1186/s12885-018-4263-3

**Published:** 2018-04-03

**Authors:** Xuan-Anh Phi, Alberto Tagliafico, Nehmat Houssami, Marcel J. W. Greuter, Geertruida H. de Bock

**Affiliations:** 1 Department of Epidemiology, University Medical Center Groningen, Hanzeplein 1, PO Box 30.001, 9700RB Groningen, The Netherlands; 2 Department of Health Sciences (Dissal), University of Genova and Ospedale Policlinico San Martino, Via L.B.Alberti 2, 16139 Genoa, Italy; 3 Sydney School of Public Health, Sydney Medical School, The University of Sydney, Edward Ford Building (A27), Sydney, NSW 2006 Australia; 4 Department of Radiology, University Medical Center Groningen, Postbus 30 001, 9700RB Groningen, The Netherlands

**Keywords:** Breast neoplasm, Digital mammography, Digital breast tomosynthesis, Review, Meta-analysis, Breast density

## Abstract

**Background:**

This study aimed to systematically review and to meta-analyse the accuracy of digital breast tomosynthesis (DBT) versus digital mammography (DM) in women with mammographically dense breasts in screening and diagnosis.

**Methods:**

Two independent reviewers identified screening or diagnostic studies reporting at least one of four outcomes (cancer detection rate-CDR, recall rate, sensitivity and specificity) for DBT and DM in women with mammographically dense breasts. Study quality was assessed using QUADAS-2. Meta-analysis of CDR and recall rate used a random effects model. Summary ROC curve summarized sensitivity and specificity.

**Results:**

Sixteen studies were included (five diagnostic; eleven screening). In diagnosis, DBT increased sensitivity (84%–90%) versus DM alone (69%–86%) but not specificity. DBT improved CDR versus DM alone (RR: 1.16, 95% CI 1.02–1.31). In screening, DBT + DM increased CDR versus DM alone (RR: 1.33, 95% CI 1.20–1.47 for retrospective studies; RR: 1.52, 95% CI 1.08–2.11 for prospective studies). Recall rate was significantly reduced by DBT + DM in retrospective studies (RR: 0.72, 95% CI 0.64–0.80) but not in two prospective studies (RR: 1.12, 95% CI 0.76–1.63).

**Conclusion:**

In women with mammographically dense breasts, DBT+/−DM increased CDR significantly (versus DM) in screening and diagnosis. In diagnosis, DBT+/−DM increased sensitivity but not specificity. The effect of DBT + DM on recall rate in screening dense breasts varied between studies.

**Electronic supplementary material:**

The online version of this article (10.1186/s12885-018-4263-3) contains supplementary material, which is available to authorized users.

## Background

Breast cancer (BC) is the most common cancer in women and the leading cause of cancer death among women in Europe [1]. Many countries have adopted population-wide BC screening, initially with film-screen and subsequently with digital mammography (DM), aiming to lower mortality from BC by earlier detection of the disease [2, 3]. However, DM has moderate sensitivity, for which estimates vary from 67.3% to 93.3% [4]. High breast tissue density, defined as having more than 50% density on mammography, categories 3 and 4 or categories c and d in the BI-RADS 4th or 5th edition respectively [5, 6] reduces the sensitivity of mammography due to its masking effect, and may increase false-positives due to superimposition of dense parenchyma. It is estimated that about half of all women taking part in screening have dense breasts although the proportion differs in age-groups [7, 8]. Breast density is also considered an independent risk factor for BC [9].

Digital Breast Tomosynthesis (DBT) enables pseudo-3D imaging of the breast, resulting in better discrimination of tissue structures and potentially improved visualisation of cancer [10, 11]. Hence, DBT has the potential to improve both sensitivity and specificity of imaging in BC screening, leading to more detected cancers with fewer false-positives [10]. However, using both DM and DBT increases radiation dose to the breast, if both acquisitions are obtained. Improved screening accuracy using DBT has been shown in several prospective and retrospective studies, in screening populations and in studies using BC-enriched mammogram series [12, 13]. Very few reviews have examined the role of DBT in women with dense breasts, and these were either concise reports or did not use systematic methodology [14, 15]. Therefore, in this work we aimed to systematically review the literature on the accuracy of DBT compared to DM in women with dense breasts. A secondary objective was to perform a meta-analysis on four outcomes (cancer detection rate - CDR, recall rates, sensitivity and specificity) of DBT compared to DM in women with dense breasts.

## Methods

A systematic review and meta-analysis were performed by two independent reviewers (XAP and GHdB or AT), following a predetermined review protocol based on the PRISMA guidelines (http://www.prisma-statement.org/) (Additional file 1). Discordance throughout the process was discussed between the two reviewers and if consensus was not reached then a third reviewer (GHdB or NH) was consulted.

We searched for studies that included women older than 18 years, who underwent breast imaging using DBT and DM and were classified as having dense breasts on mammography. Studies comparing DBT to DM and reporting at least one accuracy measure were considered. Prospective as well as retrospective comparative studies could be included.

### Data sources and searches

**Fig. 1:**
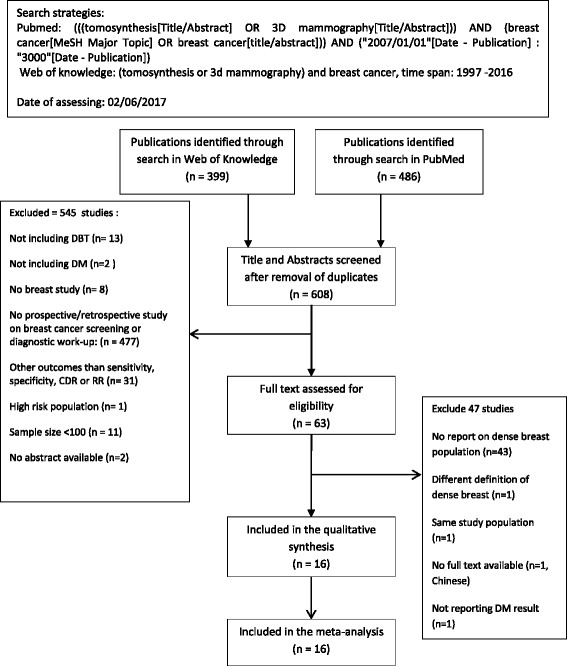
Flow-chart of study inclusion

PubMed and the Web of Science were searched for relevant English-language articles published between January 2007 up to and including May 2017. Additionally, references of identified eligible articles and reviews were manually screened for additional relevant sources. The search strategy included three main key words: Tomosynthesis or 3D mammography in the title or abstract and BC in all fields (see Fig. 1).

### Eligibility criteria

Eligible studies were: studies that compared the accuracy of DBT and DM in a screening setting or diagnostic setting; reported data on at least one of 4 outcomes (CDR, recall rate, sensitivity and specificity) for both DBT and DM (where data reported or could be calculated); included at least 100 women with dense breasts who were asymptomatic (screening setting) or recalled after screening (diagnostic setting); and where ‘dense breast’ was defined as more than 50% density [BI-RADS 3 and 4 (4th edition) or BI-RADS c and d (5th edition)]. Only English publications were considered. Studies which did not contain original data, or simulation studies, were excluded. If multiple publications were based on the same study population, the most extensive study in terms of data reported was chosen.

### Study selection

Articles identified from the search were loaded into RefWorks (2016, ProQuest LLC) and duplicates were removed. Titles/ abstracts, followed by full text, were reviewed based on predefined criteria and a final set of eligible studies were selected.

### Data collection process

A predefined form was developed, and used to extract information from included studies: type of study (prospective or retrospective), study setting (screening or diagnostic), number of women with dense breasts, inclusion and exclusion criteria, age of women with dense breast (age of whole study population if not specified for dense breasts), number of screening rounds if applicable, length of follow-up, method of reporting breast density, number of BCs, reading protocol (single or double reading), definitions for recall and for positive test, DBT manufacturer, number of DBT views (one or two), utilisation of additional modalities (DM or none), and reported outcomes for DBT and for DM in women with dense breasts (CDR, recall rate sensitivity and/or specificity).

### Risk of bias and quality appraisal

The quality of included studies was assessed using the QUADAS-2 tool which was modified to ensure assessment was appropriate for the breast screening or diagnostic context. The domains considered were: patient selection, index test, reference standard, flow and timing and applicability. This was performed by two reviewers independently and final quality assessment was based on consensus.

### Data analysis

Meta-analysis was performed to estimate the relative risk of cancer detection and of recall for DBT and DM using a random effects model in RevMan 5.3. This analysis was performed separately for screening and diagnostic studies, and also separately for studies comparing two groups of women (unpaired data) and those comparing detection within one group of women (paired data). Subgroup analyses were carried out to examine the effect of covariates, modality (whether stand-alone DBT, or DBT with DM), outcome definitions, and reading protocol (single or double-reading). A summary ROC was produced for DBT and DM for sensitivity and specificity where studies reported these outcomes. For computation, it was assumed that all screens were independent, even if there were multiple screens for some patients in some studies. Heterogeneity across studies was quantified with I^2^ measure for CDR and recall rate.

## Results

### Study inclusion

A total of 608 unique studies were eligible for title and abstract screening, and 63 studies were checked at full-text reading (details in Fig. 1). Sixteen studies [12, 16–30] met our predefined inclusion criteria and were included in the evidence synthesis. The meta-analysis was performed separately for 5 diagnostic studies, and for 11 screening studies (these were examined separately for 8 screening studies that used two independent study groups, and the 3 screening studies that used one study group). Details about study inclusion with reasons for exclusion are described in the flow-chart (Fig. 1).

### Overview of included studies

Characteristics of 16 included studies are presented in Additional file 2. Studies differed in terms of study setting, threshold definitions, breast density categorization, reading protocol and whether DBT was used alone or with DM. Among the five diagnostic studies, four studies using DBT and DM reported sensitivity and specificity [12, 18, 21, 30] and one study reported recall rate [17]. It was possible to calculate CDR from three studies which reported sensitivity and specificity [12, 18, 21]. All but two of 11 screening studies performed one screening round. Nine screening studies reported CDR [16, 19, 20, 23–27, 29] and nine studies reported recall rate [16, 19, 20, 22, 24, 26–29].

### Quality assessment

Six of the 16 included studies were at high risk of bias in terms of patient selection. In five studies, DBT was performed more often in women with dense breast, with family history of BC or it was performed based on availability and women’s preferences [20, 22, 27–29]. Three studies did not specify density classification [22, 26, 28]. Three of 16 studies were at high risk of bias in terms of index test due to unspecified outcome definition [26, 28, 29].^.^ One study did not specify the reference standard for negative images [21]. One third of the studies were at high risk of bias regarding flow and timing domain because DBT and DM were performed in different periods of time [24–27, 29]. Overview of risk of bias and applicability is shown in Table 1.

**Table 1:**
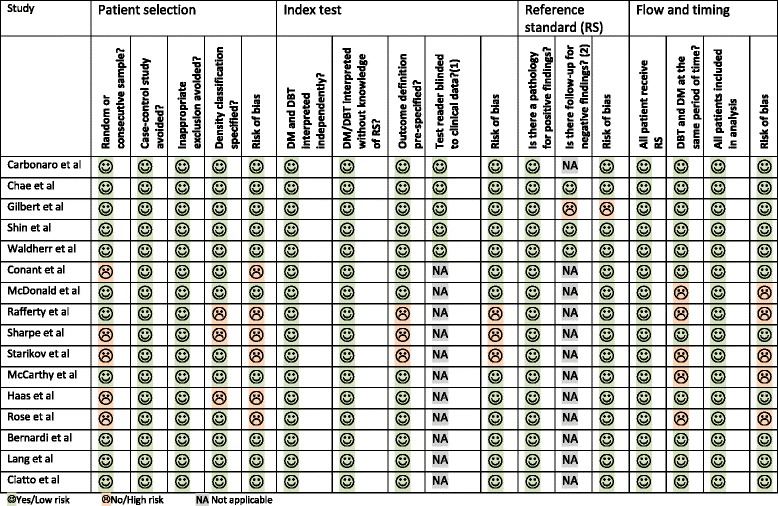
Quality appraisal of included studies

*DM* digital mammography, *DBT* digital breast tomosynthesis

(1)This question is applicable for retrospective diagnostic study. (2) This question is applicable for studies reporting sensitivity and specificity

### Comparing tomosynthesis and digital mammography in women with dense breasts in diagnostic settings (*N* = 5)

**Fig. 2:**
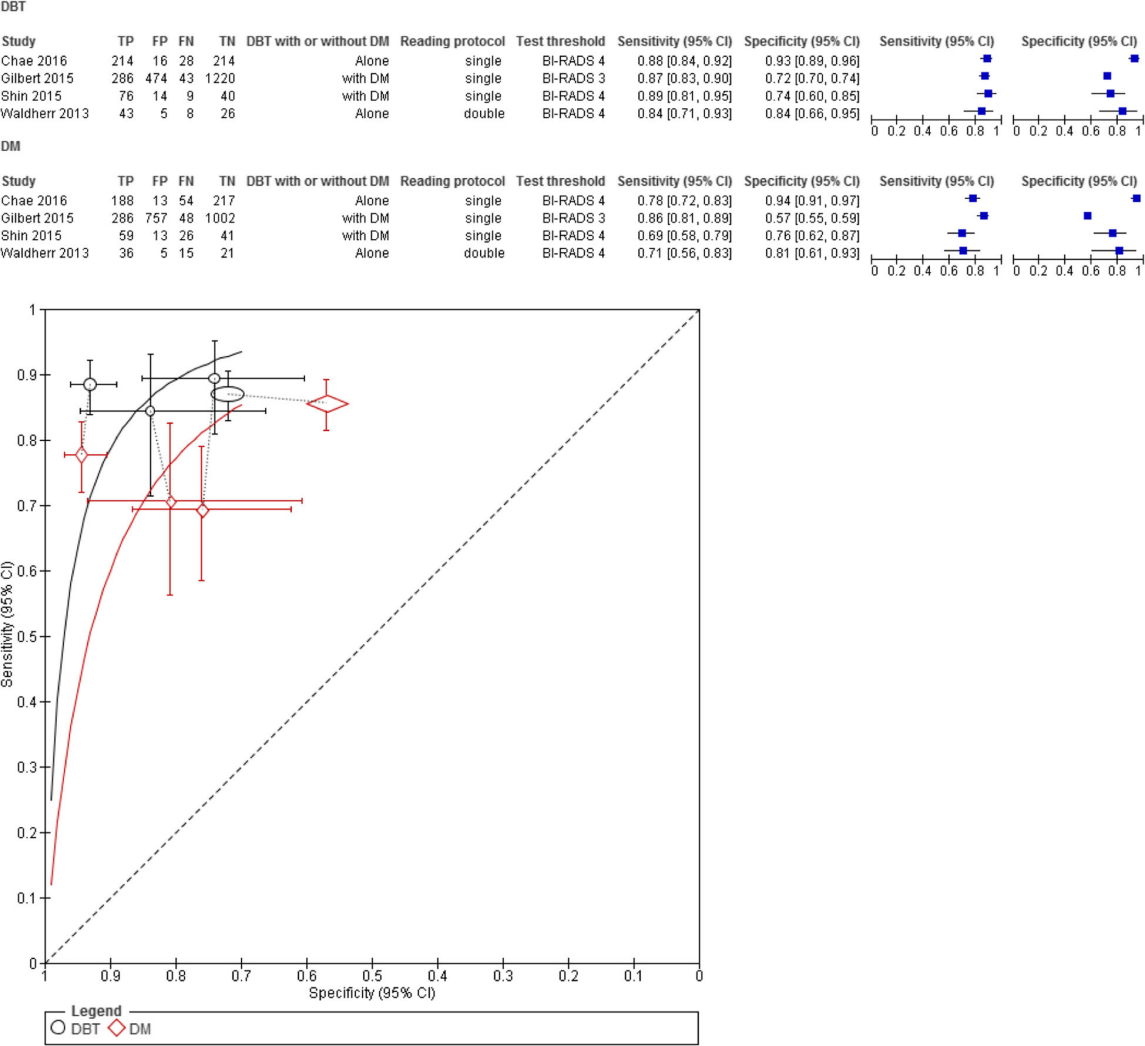
Forest plot and Summary receiver operating characteristic plot of DBT and DM in diagnostic setting

**Fig. 3:**
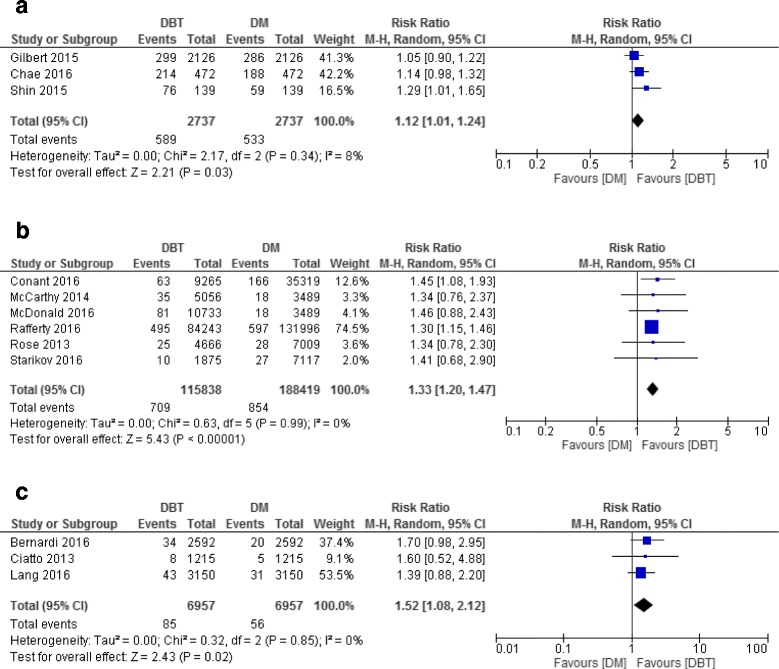
Cancer detection rate of DBT and DM in diagnostic and screening studies. **a** In diagnostic studies. **b** In screening studies using two study groups. **c** In screening studies using one study group

In the diagnostic setting, sensitivity of DBT ranged from 84% (95% CI 71–93) [30] to 89% (95% CI 81–95) [12], being higher than the sensitivity of DM which ranged from 69% (95% CI 58–79) [12] to 86% (95% CI 81–89) [21] (Fig. 2). The specificity of DBT ranged from 72% (95% CI 68–72) [21] to 93% (95% CI 89–96) [18] and was not different from the specificity of DM which ranged from 57% (95% CI 55–59) [21] to 94% (95% CI 91–97) [18] (Fig. 2). Using DBT with or without DM improved CDR with a ratio of 1.12 (95% CI 1.01–1.24) compared to DM alone (Fig. 3a). Heterogeneity across studies was small (I^2^ = 8%). Only one study reported a significant reduction in recall rate using DBT plus DM compared to DM alone (RR = 0.56, 95% CI 0.47–0.66) [17]. Due to the small number of studies (*n* = 5), subgroup analysis was not performed.

### Comparing tomosynthesis and digital mammography in women with dense breasts in screening setting

#### Studies comparing two groups of participants (*N* = 8)

**Fig. 4:**
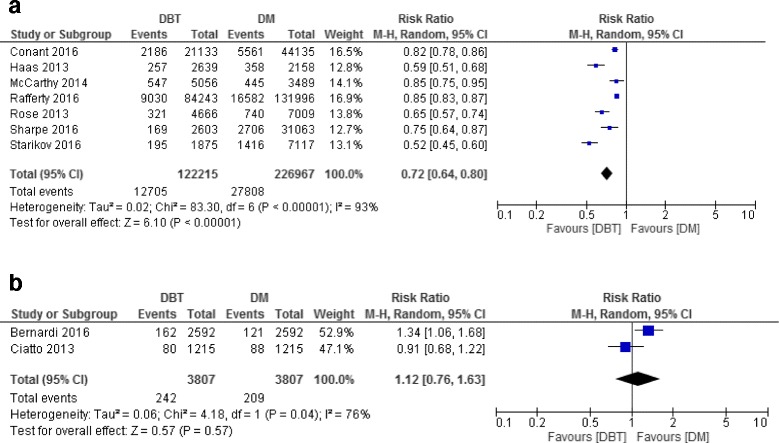
Recall rate of DBT and DM in screening studies. **a** Studies used two study groups. **b** Studies used one study group

All but two studies used single-reading. Six studies were included in the analysis of CDR and were homogeneous in reporting CDR (I^2^ = 0%). CDR was estimated to be significantly higher when using DBT with or without DM compared to DM alone (RR = 1.33, 95% CI 1.20–1.47) (Fig. 3b). Seven studies reported recall rates with high heterogeneity (I^2^ = 93%). Pooled estimate for these studies showed a significant reduction in recall rate when using DBT with or without DM compared to DM alone (RR = 0.72, 95% CI 0.64–0.80) (Fig. 4a). Subgroup analysis based on test definition showed consistently reduced recall rates (RR = 0.59, 95% CI 0.52–0.67 for BI-RADS 0 as recalled and RR = 0.84, 95% CI 0.81–0.87 for other definitions). Subgroup analysis also reduced heterogeneity in subgroup estimates (I^2^: 62% and 37%, respectively) (Additional file 3).

#### Studies comparing within the same group of participants (paired data) (*N* = 3)

These studies used DBT combined with DM [16, 19] or DBT alone [23], double-reading protocol and similar definitions for recall. Pooled estimates from three studies showed improved CDR when using DBT with DM compared to DM alone, RR = 1.52, 95%CI 1.08–2.12 (Fig. 3c) with homogeneity across studies (I^2^ = 0%). Using DBT with DM did not reduce recall rate based on two studies (I^2^ = 76%). The pooled RR was 1.12, 95% CI 0.76–1.63 (Fig. 4b). Subgroup analysis was not performed due to a small number of studies.

## Discussion

This systematic review identified 16 studies (5 diagnostic and 11 screening studies) comparing accuracy measures, such as CDR, recall rate, sensitivity and specificity, of DBT and DM in women with dense breasts at mammography. We found that in diagnostic studies, DBT with or without DM improved CDR (RR = 1.12, 95% CI 1.01–1.24) and sensitivity compared to DM alone (84%–89% vs 69%–86%) in women with dense breasts, whereas specificity did not increase when DBT was used (72–93% vs 57–94%). In the screening setting, CDR was improved when using DBT with or without DM, in studies comparing within one study group (RR = 1.52, 95% CI 1.08–2.12) or comparing two study groups of participants (RR = 1.33, 95% CI 1.20–1.47). Recall rate was reduced when using DBT compared to DM alone in screening studies using two study groups (RR = 0.72, 95% CI 0.64–0.80), though heterogeneity across studies was very high (I2 = 93%) and partially explained by the two different definitions of outcome.

Almost all of the reviews in the literature comparing DBT with DM in BC screening do not distinctly report on women with dense breasts. One review, not restricted to women with dense breasts, reported that DBT with DM increased CDR with a RR of 1.29 (95% CI 1.16–1.43) (Yun et al. [31]) which is comparable to our estimate. We identified only two reviews reporting on women with dense breasts, one was a quantitative rapid review and one was a narrative review without analyses [14, 15]. The rapid review identified eight studies comparing CDR and recall rate of DBT plus DM to DM alone in women with dense breasts but was restricted to screening studies. The rapid review reported a significantly increased CDR when pooling studies comparing within same group of participants (incremental cancer detection per 1000 screens: 3.9, 95% CI 2.7–5.1) as well as when pooling studies comparing two groups of participants (incremental cancer detection per 1000 screens: 1.4, 95% CI 0.9–2.0) [14]. Although our results are generally in line with these previous reviews, by performing a systematic search and by considering both screening and diagnostic studies, we were able to identify more data sources for the comparison of DBT with DM, and were also able to present data on a broader range of outcomes (sensitivity and specificity as well as CDR and recall rate) hence we extend on existing reviews. In addition, we conducted quality assessment of the included evidence which was not done in the other reviews on this issue.

Studies included in our review were heterogeneous in several aspects. Firstly, some studies included asymptomatic or symptomatic population. Although aiming to investigate the accuracy of DBT and DM in BC screening, some studies included women who were recalled after screening [12, 17, 21, 30]. By doing so, they obtained populations recalled to assessment which have higher cancer rates than unselected asymptomatic populations. However, the results from the screening and diagnostic settings had generally comparable findings. Secondly, retrospective studies tended to perform single-reading whereas the prospective studies performed double-reading (reflecting screening practice in various settings) which may increase CDRs in the latter. In the two STORM trials, screen-reading results were based on recall by either reader, making the recall rate of integrated DM and DBT higher than DM in STORM-2 [16] and non-significantly lower in STORM-1 [19]. Additionally, all but one [23] screening study used DBT together with DM while among five diagnostic studies two studies used DBT as stand-alone modality [17, 30].

Another difference among studies was the outcome definitions which may be contributing to some of the observed heterogeneity. Studies performed in the United States used the BI-RADS system for reporting recall whereas the European studies used a simplified ‘recall or no recall’ reporting for screen-readings. Amongst studies using BI-RADS, different thresholds were used to define recall or positive test. When analysing data for different thresholds, the result of recall rate in screening studies using two study groups remained significantly lower for DBT compared to DM but the heterogeneity decreased. Studies defining BI-RADS 0 as recall [22, 27, 29] showed a larger decrease in recall rate than studies using a different definition (BI-RADS 0,3,4,5 [20] or BI-RADS 0,4,5 [25] or where unspecified [26, 28]). Among four diagnostic studies reporting sensitivity and specificity, one study from the UK [21] used a lower threshold (BI-RADS 3 instead of BI-RADS 4) and reported lower specificity than other diagnostic studies which may account for more false positives.

The main limitation of the data used in our analysis is that all but two screening studies [20, 23] used only a single screening (likely to be first round) for DBT. When only first DBT screening rounds are used [19, 24, 26–29], more prevalent cases are usually detected, increasing CDR and potentially exaggerating the contribution of DBT to screen-detection measures. Another limitation is the short temporal perspective in all these studies: because of the recent introduction of DBT, the lack of long follow-up makes it impossible to assess whether the improved CDR and sensitivity of DBT screening further reduces BC mortality through screening compared to screening with DM alone. The retrospective studies had one major limitation in that they used two study groups (DM group versus DBT group) which were not randomly assigned to screening modalities. The study groups were from different time periods (or services) and in the DBT implementation phase, or due to the limit of DBT availability, there may have been selection to DBT screening and hence potential bias. In those studies, DBT groups were more likely to include women with dense breasts and family history indicating high cancer rate. The incremental value of DBT in those studies may be partially due to possible selection bias. Finally, in order to be able to compute the results, we made an assumption of independent screens, which might not be the case in the few studies that included more than one screening round [20, 25] or where study populations might overlap [16, 19]. However, estimates from those studies were similar to the other studies, thus we do not foresee that this assumption affected our reported findings.

We performed a systematic review to summarize current evidence on the use of DBT in BC screening and diagnosis specifically in women with dense breasts on mammography. We identified and systematically examined data for women with dense breasts from 16 eligible studies to report the most extensive review so far on the accuracy of DBT in women with dense breasts. Moreover, this is the first review assessing the quality of evidence and bias in the studies on DBT in women with dense breasts.

## Conclusion

We found that in both the screening and diagnostic settings, DBT improved CDR (versus DM) in women with dense breasts. In the diagnostic setting, using DBT with or without DM increased sensitivity but did not change specificity. There was a significant reduction in recall rate when using DBT with DM (versus DM) in retrospective screening studies comparing between two study groups, although heterogeneity across studies was relatively high. A small number of prospective studies conducted in organized screening programs did not show reduced recall from using DBT. Improved CDR and reduced recall rate from DBT may imply a more effective screening test or diagnostic work-up for women with dense breasts. However, the critical issue is that more studies with longer follow-up and more screening rounds are necessary to draw definite conclusions on whether this improvement in cancer detection has an impact on interval cancer rates and potentially on BC mortality.

## Additional files

Additional file 1: Prisma 2009 checklist. (DOC 62 kb)

Additional file 2: Overview of included studies (order sorted by study setting and year of publication). (DOCX 27 kb)

Additional file 3: Subgroup analysis - Recall rate of DBT and DM in screening studies using two study groups by outcome definition. (DOCX 30 kb)

## Abbreviations

BC: Breast cancer
BI-RADS: Breast Imaging Reporting and Data System
CDR: Cancer detection rate
DBT: Digital breast tomosynthesis
DM: Digital mammography
RR: Relative risk
