# Supplementary material for: Digital breast tomosynthesis for breast cancer screening and diagnosis in women with dense breasts – a systematic review and meta-analysis
Source: BMC Cancer. 2018 Apr 3;18:380. doi: 10.1186/s12885-018-4263-3 (PMC5883365; doi:10.1186/s12885-018-4263-3)
Supplement: Supplementary file 2 — Overview of included studies (order sorted by study setting and year of publication). (DOCX 27 kb) [file 12885_2018_4263_MOESM2_ESM.docx]

**Additional file 2: Overview of included studies (order sorted by study setting and year of publication)**

| **Study, year of publication,**  **Country** | **Study design**  **N of rounds**  **Duration of follow-up for negative cases**  **Age*** | **Inclusion and exclusion criteria** | **Breast density classification,**  **N of women with dense breasts,**  **N of screens,**  **N of breast cancers (BCs)** | **Reported outcomes,**  **Definition of dense breasts** | **Comparison, including number of views,**  **Reading protocol,**  **Manufacturer** |
| --- | --- | --- | --- | --- | --- |
| **Diagnostic setting studies** | | | | | |
| 1. Carbonaro et al (2016)  Italy | Prospective single cohort study  N of rounds: 1  Follow-up: median 2 years (range 1-3)  Age: mean 55, range 45-74 | Included: women recalled from the screening program and afferent to the involved institutions for diagnostic work-up  Excluded: lack of written consent, symptomatic women, pregnant women and women with breast implant in the breast with a suspicious finding | BI-RADS edition: 5  N of women: 142 women  N of screens: NS  N of BCs: NS | Recall rate: recall by either screen reader of double reading | Two view DBT+DM versus two view DM,  Double reading,  DBT, DM: Giotto Tomo (IMS) |
| 1. Chae et al 2016   Korea | Prospective single cohort study  N of rounds: 1  Follow-up: 1 year  Age: mean 49, range 30-75 | Included: women with suspected BC (BI-RADS 4,5) or newly diagnosed with BC (BI-RADS 6)  Excluded: women younger than 30 years old, women undergoing prior excisional biopsy or mammotome excision, women with breast implants | BI-RADS edition 4  472 breasts  N of BCs: 242 (including screen detected BCs) | Sensitivity/specificity: defined BI-RADS 4,5 as positive test | One-view DBT vs two-view DM  Single reading  DM Senograph DS (GE)  DBT: Senograph DS prototype (GE) |
| 1. Gilbert et al (2015)   United Kingdom | Retrospective single cohort study  N of rounds: 1  Follow-up: NS  Age: mean 56, range 29-85 | Included: women aged 47–73 years recalled to an assessment clinic for a mammographic abnormality detected at routine breast screening and women below 50 years of age with a family history of breast cancer who attended annual mammography.  Excluded: women with breast implants, who were, and who were unable to give inform consent | Density percentage ≥ 50%  N of women: 2126  N of BCs: 334 BCs (including screen detected BCs) | Sensitivity/specificity: defined BI-RADS 3,4,5 to be positive test | Two-view DBT+DM vs DM  Single reading  DM, DBT: Selenia Dimensions (Hologic) |
| 1. Shin et al (2015)   Korea | Retrospective single cohort study  N of rounds: 1  Follow-up: mean 1.8 ± 0.11  Age: mean 50, SD 10.4 | Included: women with clinical symptoms and those referred for diagnostic work-up, and who showed an abnormality at screening mammography or ultrasound  Excluded: women with previous breast surgery, history of neoadjuvant chemotherapy, multifocal BC in both breasts | BI-RADS edition 5  N of women: 139  N of BCs: 85 (including screen detected BCs) | Sensitivity/ specificity: defined BI_RADS 4,5 for positive test | One-view DBT+DM vs two-view DM  Single reading  DM,DBT:Selenia Dimensions (Hologic) |
| 1. Waldherr et al (2013)   Switzerland | Retrospective single cohort study  N of rounds: 1  Follow-up: 12-16 months  Age: NS | Included: women recalled from screening and those with symptoms | BI-RADS edition 4  N of women: NS  N of BCs: NS | Sensitivity/specificity: defined BI_RADS 4,5 for positive test | One-view DBT vs two-view DM  Double reading  DM: NS  DBT: Selenia prototype (Hologic) |
| **Screening setting studies comparing accuracy of DM and DBT between two difference populations** | | | | | |
| 1. Conant et al (2016)   The United State of America | Retrospective nonrandomized controlled study  N of rounds >=1  Follow-up: 1 year  Age: range 40-74 | Included: women aged 40-74 without a history of BC, who had no other BC screening 3 months prior to screening  Excluded: none | BI-RADS edition 4  DM: 35,319 subsequent screens, 166 detected BCs  DBT+DM: 9,265 subsequent screens, 63 detected BCs | Cancer detection: defined BI-RADS 0,3,4,5 for positive test  Recall rate: defined BI-RADS 0,3,4,5 for recall | Two-view DBT+DM vs Two-view DM  Reading NS  Manufacturer NS |
| 1. McDonald et al 2016   The United State of America | Retrospective study with historical control group  N of rounds: 4  Follow-up: 1 year  Age: mean 56.8, SD 11.0 | Included: women who underwent screening, without history of BC or clinical symptoms.  Excluded: none | BI-RADS edition 5  DM: 3,489 screens, 18 screen detected BCs  DBT: 10,733 screens, 81 detected BCs | Cancer detection: defined as histological proven cancer among recalled cases  False positive recall: NS  Dense breasts: category 3-4 | Two view DBT+DM vs two view DM  Single reading  DM, DBT: Dimensions (Hologic) |
| 1. Rafferty et al 2016   The United State of America | Retrospective study with historical control group  N of rounds: 1  Follow-up: none  DM age^&^: mean 57, range 54.4-60.5  DM+DBT age^&^: mean 56.2, range 52.6-59.7 | Included: women presenting for screening  Excluded: none | BI-RADS edition: NS  DM: 16,582 screens, 597 detected BCs  DBT: 9,030 screens, 495 detected BCs | Cancer detection : unspecified definition for test positive  Recall rate: unspecified definition for recall | Two view DBT+DM vs two view DM  Single Reading  DM, DBT: Selenia Dimensions (Hologic) |
| 1. Sharpe et al (2016)  The United State of America | Retrospective nonrandomized controlled study  N of rounds: NS  Follow-up: NS  DM age: mean 57.6, SD 10.9  DBT age: mean 55.7, SD 9.74 | Included: asymptomatic women presenting for screening  Excluded: women with breast symptoms, or diagnosed with BC within 5 years | BI-RADS edition: NS  DM: 31,063 screens  DBT: 2,603 screens  N of BCs: NS | Recall rate: unspecified definition for recall | Two-view DBT+DM vs two-view DM  Single reading  DM: Essential, DS, 2000D (GE)  DBT: Selenia Dimensions (Hologic) |
| 1. Starikov (2016)  The United State of America | Retrospective nonrandomized controlled study  N of rounds: 1  Follow-up: NS  Age: NS | Included: women present for screening  Excluded: women with history of BC, symptoms. | BI-RADS edition 5  DM: 7,117 screens, 27 detected BCs  DBT: 1,875 screens, 10 detected BCs | Cancer detection : unspecified definition for test positive  Recall rate: defined BI_RADS 0 for recall | DBT+DM vs DM  Single reading  Number of views NS  Manufacturer NS |
| 1. McCarthy et al (2014)  The United State of America | Retrospective study with historical control group  N of rounds: 1  Follow-up: 6 months  DM age: mean 56.9  DM + DBT age: mean 56.7 | Included: women presenting for screening Women with no personal history or symptoms, no clinical signs or symptoms. Women with breast implants or large breast are included.  Excluded: none | BI-RADS edition 4  DM: 3,489 screens, 18 screen detected BCs  DM+DBT: 5,056 screens, 35 screen detected BCs | Cancer detection: defined as histologically proven cancers among recalled cases within 180 days.  False positive recall: NS  Dense breasts: category 3-4 | Two view DBT+DM versus two view DM  Single reading  DM, DBT: Dimensions (Hologic) |
| 1. Haas et al (2013)  The United State of America | Retrospective nonrandomized controlled study  N of rounds: 1  Follow-up: NS  DM age: mean 57.5  DBT age: mean 55.8 | Included: women presenting for screening between 10/2011 and 9/2012  Excluded: women with breast implants, or large breast requiring tiled images | BI-RADS edition NS  DM: 2158 women  DBT: 2639 women  N of BCs: NS | Recall rate: defined BI-RADS 0 for recall | Two view DBT+DM vs two view DM  Reading NS  DM, DBT: Selenia Dimensions (Hologic) |
| 1. Rose et al (2013)   The United State of America | Retrospective with historical control group study  N of rounds: 1  Follow-up: NS  Age NS | Included: asymptomatic women from age 18 years old, self-selected to screening. Include images read by radiologist who had read with at least 500 mammograms.  Excluded: none | BI-RADS edition 4  DM: 7,009 screens, 28 detected BCs  DBT: 4,666 screens, 25 detected BCs | Cancer detection: defined BI-RADS 0 for positive test  Recall rate: defined BI-RADS 0 for recall | DBT+DM vs DM  Single reading  Unspecified number of views  DM: Selenia (Hologic)  DBT: Dimensions (Hologic) |
| **Screening setting studies comparing accuracy of DM and DBT in the same women** | | | | | |
| 1. Bernardi et al 2016   Italy | Prospective study  N of rounds: 1 (95% of the screens were repeat screens)  Follow-up: none  Age: median 58 (IQR 53-63) | Included: asymptomatic women aged 49 or older, at standard (population) risk for BC attending biennial screening mammography through the Trento screening programme  Excluded: not specified | BI-RADS edition 5,  2592 women,  2592 screens  36 screen detected BCs | Cancer detection : defined as histological proven cancers among recalled cases,  False positive recall: Recall without cancer,  Dense breasts: category 3-4 | Two view DM, versus two view DBT+DM versus two view DBT and synthetic DM,  Double reading,  DM, DBT: Selenia Dimensions (Hologic ) |
| 1. Lang et al (2016)    Sweden | Prospective study  N of rounds: 1  Follow-up: ≥1 year  Age: mean 56, range 40-76 | Included: women age 40-74 are invited to the screening program  Excluded: pregnant women, not speaking Swedish or English | BI-RADS edition 5  3150 examinations  68 screen detected BCs | Cancer detection: defined recalled cases as positive test. Recall was defined by arbitration for two readers. | One-view DBT (MLO) vs one-view DM  Double reading  DM, DBT: Mammomut Inspiration (Siemens) |
| 1. Ciatto et al (2013)   Italy | Prospective study  N of rounds: 1  Follow-up: NS  Age: median 58, IQR 53-64, range 48-71 | Included: asymptomatic women from age 48 attended screening mammography every 2 years  Excluded: without informed consent | BI-RADS edition 4  1215 screens  8 screen detected BCs | Cancer detection : defined recalled cases as positive test  Recall rate: recall by either screen reader of the double readings | Two view DBT+DM vs two view DM  Double reading  DM, DBT: Selenia Dimensions (Hologic) |

* Reported data are related to all women participating in this study as the data were not presented for women with dense breasts separately.

& This information was not extracted directly from the included study but from previous publication on the same population.

NS: Not specified; BC: breast cancer; DM: digital mammography; DBT: digital breast tomosynthesis; vs: versus
