# Supplementary material for: Digital breast tomosynthesis for breast cancer screening and diagnosis in women with dense breasts – a systematic review and meta-analysis
Source: BMC Cancer. 2018 Apr 3;18:380. doi: 10.1186/s12885-018-4263-3 (PMC5883365; doi:10.1186/s12885-018-4263-3)

**Additional file 3: Subgroup analysis - Recall rate of DBT and DM in screening studies using two study groups by outcome definition**


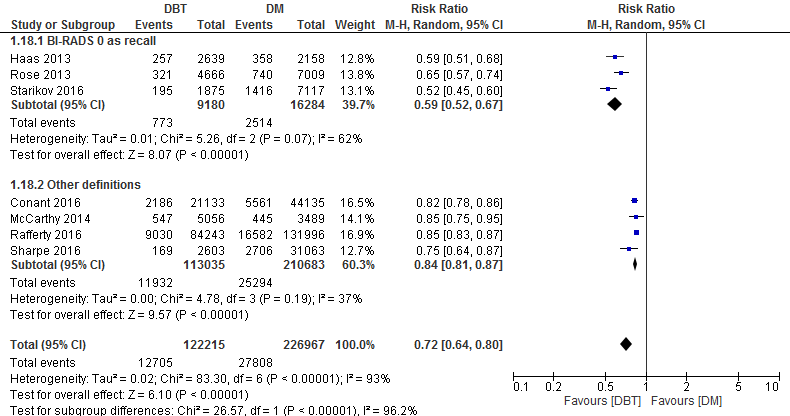

Supplement: Supplementary file 3 — Subgroup analysis - Recall rate of DBT and DM in screening studies using two study groups by outcome definition. (DOCX 30 kb) [file 12885_2018_4263_MOESM3_ESM.docx]
